# Influence of Endothelin-1 in Aqueous Humor on Intermediate-Term Trabeculectomy Outcomes

**DOI:** 10.1155/2016/2401976

**Published:** 2016-01-21

**Authors:** Lars Choritz, Benjamin Mahmoodi, Hagen Thieme

**Affiliations:** ^1^University Hospital Magdeburg, Department of Ophthalmology, Otto-von-Guericke University, Leipziger Strasse 44, 39120 Magdeburg, Germany; ^2^University Medical Center Mainz, Department of Ophthalmology, Johannes Gutenberg University, Langenbeckstrasse 1, 55131 Mainz, Germany

## Abstract

*Purpose*. To investigate whether increased concentrations of ET-1 in aqueous humor of glaucoma patients influences surgical outcome of standard trabeculectomy with Mitomycin C.* Methods*. Retrospective chart review of 36 glaucoma patients with known ET-1 concentrations who had undergone trabeculectomy with Mitomycin C. Patients were divided into two groups based on their aqueous ET-1 concentration, a below-median (low ET-1) and an above-median (high ET-1) group. Postoperative IOP development, necessity of glaucoma medication, surgical success and complications, postoperative use of antifibrotics (5-FU), and number of additional glaucoma surgeries were compared between the groups.* Results*. Overall surgical success of trabeculectomy was comparable to published literature (90%, 81%, 76%, and 68% absolute success at 12, 24, 36, and 48 months after surgery). There was no difference between high and low ET-1 group in the postsurgical development of IOP, surgical success rate, or complication rate. There was no difference in postoperative scarring or indirect indicators thereof (e.g., number of 5-FU injections, needlings, suture lyses, or IOP lowering medications).* Conclusion*. In this set of patients, ET-1 in aqueous humor does not appear to have influenced surgical outcome of trabeculectomy with Mitomycin C. There is no indication of an increased likelihood of bleb fibrosis in patients with increased ET-1 concentrations.

## 1. Introduction

Endothelin-1 (ET-1), one of the most potent vasoconstrictors known, has been shown to be produced in the eye by the ciliary epithelium and to be released into the aqueous humor [1, 2]. The two endothelin receptors (ETAR and ETBR) are expressed by various ocular tissues, including ciliary and iris muscles, trabecular meshwork, cornea, vasculature, and astrocytes in the optic nerve head [3–6]. Despite this apparent ubiquity of endothelin signaling, the peptide's physiological role in the eye is poorly understood.

ET-1 has also been implicated in the pathophysiology of glaucoma. Several studies showed increased concentrations of ET-1 in the aqueous humor of patients with different forms of glaucoma and pleiotropic effects of the peptide on various ocular tissues. Consequently, ET-1 antagonists have been suggested as therapeutic principle for glaucoma [7].

In the anterior segment, ET-1 is believed to be involved in the regulation of intraocular pressure (IOP) via effects on the contractility of ciliary muscle and trabecular meshwork. Pharmacological studies on native bovine trabecular meshwork suggest that an increased contractility of the trabecular meshwork with subsequent reduction of intertrabecular spaces leads to increased outflow resistance and IOP [8, 9]. Evidence is inconclusive, however, as injection of ET-1 into intact eyes caused both increases and decreases of IOP depending on ET-1 concentration and animal species. In rabbits, for instance, injection of ET-1 into the anterior chamber caused a biphasic response: an initial increase in IOP for up to 2 hours, followed by a sustained decrease in IOP for up to 72 hours [10]. These differential effects are most likely related to differential actions of the two ET receptors, as ET-AR antagonists effectively abolished IOP increase but not IOP decrease [11], while ET-BR agonists caused only IOP decrease without prior IOP increase [12]. Interestingly, damage to the trabecular meshwork of rabbits induced by Argon Laser Trabeculoplasty (ALT) leads to a transient increase in ET-1, followed by an increase in IOP that could be inhibited by an ET-AR antagonist as well [13, 14]. A similar IOP spike is often seen in humans after ALT [15] and might also be caused by ET-1 release [13], which is why only patients with no prior ocular surgery or trauma were included in the initial study [16].

More recently, Wang et al. showed that topical administration of a nonpeptide ETAR-antagonist (SPP 301) caused sustained, dose dependent IOP reductions in glaucomatous monkey eyes, giving creed to the notion of a regulatory role for ET-1 in IOP control [17].

In humans, evidence for contractility of the trabecular meshwork (TM) is scarce and indirect. Presence and functionality of a smooth muscle-like contractile apparatus have been shown by histological and molecular biological methods [18]. Pharmacologically induced contraction (e.g., by ET-1) of cultured human TM cells embedded in collagen gels implies that TM contractility may contribute to the physiological regulation of IOP [19, 20]. However, direct contraction of native human TM in response to ET-1 has yet to be shown. Moreover, despite the early finding of increased ET-1 in aqueous samples of glaucoma patients, a statistically significant correlation between ET-1 in aqueous and IOP just prior to sample acquisition was established only recently [16]. Much work is needed, to fully understand the complex actions of ET-1 in the eye in general, and its role in regulating IOP in particular.

ET-1 has also been shown to contribute to fibrosis and fibrotic disorders in various organ systems, including lung, skin, liver, kidney, heart, and vasculature. Several studies have shown ET-1 to facilitate transdifferentiation of resident fibroblasts as well as epithelial/endothelial cells, hepatic stellate cells, or monocytes into myofibroblasts, a predominant cell type in fibrotic disorders (for review see [21]). Since failure to control IOP after trabeculectomy is most often the result of excessive scarring, understanding the underlying mechanisms of bleb fibrosis is of major interest for the further improvement of success rates of filtering surgery. So far ET-1 has not been studied as a potential modulator of wound healing after glaucoma surgery.

The aim of this retrospective chart review therefore was to investigate whether increased concentrations of ET-1 in the aqueous samples of glaucoma patients influenced wound healing and bleb fibrosis after standard trabeculectomy with MMC. Secondly, postsurgical IOP development was assessed in order to elucidate whether bypassing the trabecular meshwork offsets the previously observed correlation between ET-1 and IOP.

## 2. Methods

### 2.1. Study Design/Patient Selection

This investigation was conducted as retrospective chart review of 36 patients who had previously been enrolled in a study to determine whether there was a correlation between ET-1 in aqueous humor and IOP. In this previous study aqueous humor samples from a total of 94 patients with either cataract (control), primary open angle glaucoma (POAG), or pseudoexfoliation glaucoma (PEXG) were collected during routine cataract surgery or trabeculectomy. ET-1 concentrations in the samples were determined by a subtype-specific ELISA and correlated with the last presurgical IOP (as determined by Goldman applanation tonometry). Written consent was obtained. Approval by the local ethics committee was granted (App. number 837.198.06 [5296]), and all tenets of the Declaration of Helsinki were observed.

Patient selection for the previous study was very rigid. In order to avoid any confounding conditions, all patients with ophthalmological disorders other than cataract, POAG, or PEXG were excluded. All eyes were naïve to any surgical intervention. Furthermore, all systemic conditions that are known to be associated with systemic changes to ET-1 concentration (e.g., cardiovascular diseases, diabetes mellitus, pulmonary diseases, and solid tumors) were also excluded. Thus, the patients included did not comprise a typical sample of the glaucoma or cataract population, with regard to typical comorbidity with other age-related diseases. For details on the patient selection and its rationale, see [16].

Of the 94 patients with known ET-1 concentrations in aqueous humor from the previous study, 56 were glaucoma patients. Of these, hospital records for at least three follow-up visits after initial discharge were on file for 53 patients. For the purpose of this investigation we chose all 36 records of the patients, who had undergone trabeculectomy during the initial study (25 POAG, 11 PEXG). Aqueous samples of the remaining 17 glaucoma patients were obtained during cataract surgery in the initial trial.

These 36 patients were divided into two groups based on their previously determined ET-1 aqueous concentration into an above-median (high ET-1, *n* = 18) and a below-median (low ET-1, *n* = 18) group. For subgroup analysis, both POAG and PEXG patients were subdivided by the same principle.

Importantly, the ophthalmologists performing follow-up visits were masked to the ET-1 concentrations obtained during the initial trial. Examination results and clinical decisions were therefore not biased.

### 2.2. Criteria for Surgical Success

Based on AGIS criteria and WGA guidelines for designing and reporting surgical success in glaucoma trials trabeculectomy was considered an “absolute success,” if IOP was below 18 mmHg and IOP reduction >20% at all postsurgical follow-up visits without additional medication or the need for further surgical intervention. Patients requiring adjunct pressure lowering medication to reach 18 mmHg or who needed early revision surgery for complications were considered “relative success.” Patients, whose IOP was not below 18 mmHg during one or more follow-up visits, or who needed late bleb revision due to fibrosis or additional pressure lowering surgery, were considered as “failure.”

### 2.3. Statistical Analysis

All data are presented as mean ± standard deviation, unless indicated otherwise. All statistical analyses were performed using PASW (SPSS version 18) software (SPSS Inc.) or GraphPad Prism (Version 5, GraphPad software Inc.). Since the sample size in this study was relatively small and the investigation was exploratory in nature, no correction for multiple testing was employed. *p* values for group comparisons were calculated only as means of identifying potential parameters of interest for future confirmatory studies.

## 3. Results

### 3.1. Patient Characteristics before Surgery

Of the 36 patients investigated for this study, 25 had undergone trabeculectomy for POAG and 11 patients had PEXG. There was no difference with regard to age, gender, study eye, number of glaucoma medications, or follow-up time between these patients. The overall characteristics of the study patients as well as their last IOP before surgery (as measured by Goldmann applanation tonometry) and the ET-1 concentration in the aqueous samples collected during surgery are displayed in Table 1. As shown in the previous study, from which these patients were selected, there was a significantly higher IOP and a higher ET-1 concentration in aqueous in the PEXG group.

Based on their known ET-1 concentrations, all patients were divided into an above-median (high ET-1) and a below-median (low ET-1) group. As displayed in Table 2, the two groups showed no relevant differences apart from ET-1 concentration (the selection criterion) and IOP, the latter of which was to be expected based on the correlation between ET-1 and IOP that was found in the previous study. Notably more of the PEXG patients were in the high ET-1 group; this difference was not significant, however, and also to be expected based on the higher mean ET-1 concentration in this group.

### 3.2. IOP Development after Surgery

**Figure 1:**
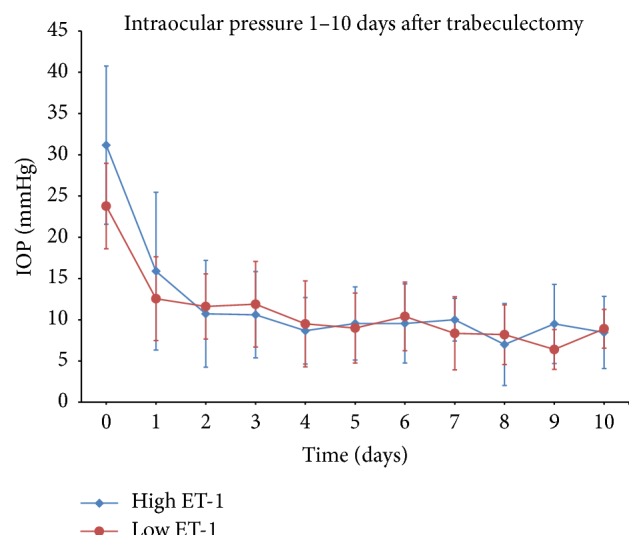
Mean intraocular pressure before and after trabeculectomy. The initial statistically significant difference in IOP, which is likely linked to the difference in ET-1 concentration, is quickly abolished after surgery. There is no difference in IOP after trabeculectomy.

**Figure 2:**
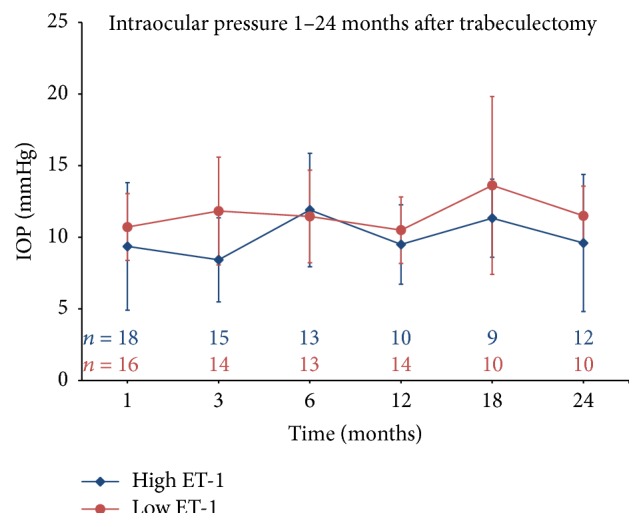
Long-term development of IOP up to 24 months after surgery. There is no significant difference between the groups.

IOP was well controlled in almost all patients after surgery. There was no difference between IOP development between patients with high and those with low ET-1 concentrations in aqueous, both in the immediate follow-up period (up to ten days after surgery, Figure 1) and up to 24 months after surgery (Figure 2). Similarly, there was no difference between POAG and PEXG patients. The difference in IOP before surgery was quickly abolished after surgery and low pressure was maintained during long-term follow-up (data not shown).

### 3.3. Adjunct Medication and Interventions

**Figure 3:**
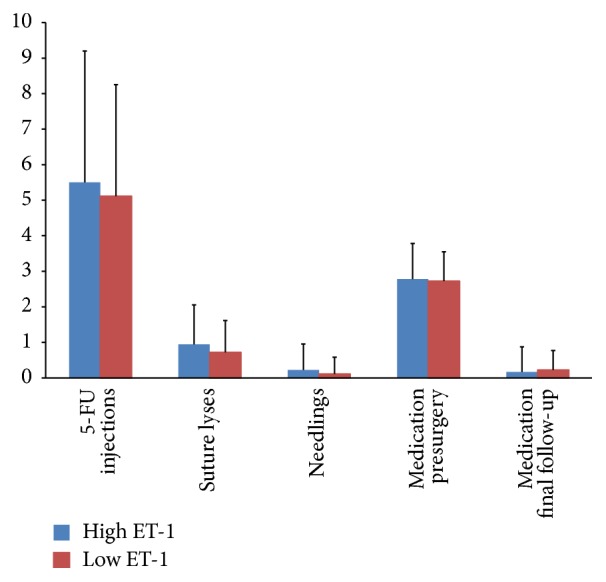
Comparison of different parameters indirectly indicative of fibrosis or failure to control IOP at the last follow-up visit. There is no difference between the high ET-1 group (blue bars) and the low ET-1 group (red bars) for any of the recorded parameters.

At final follow-up visit on record, there was no difference in the average number of glaucoma medications needed to maintain a sufficiently low IOP. The high ET-1 group required 0.18 ± 0.71 medications versus 0.22 ± 0.55 medications in the low ET-1 group (*p* = 0.794, Figure 3). All of the additional medications were used in POAG patients (0.3 ± 0.7), whereas the PEXG patients required none. This difference was not statistically significant (*p* = 0.220).

The number of postsurgical interventions was similar between patients with high and low ET-1 concentrations. The high ET-1 group required 5.5 ± 3.7 5-FU injections, 0.9 ± 1.1 suture lyses, and 0.2 ± 0.7 needlings, whereas the low ET-1 group had 5.1 ± 3.1 5-FU injections, 0.7 ± 0.9 suture lyses, and 0.1 ± 0.5 needlings (*p* = 0.736, *p* = 0.513, and *p* = 0.592, resp.; Figure 3). Comparison between POAG and PEXG showed a similar picture with 5.6 ± 3.7 versus 4.6 ± 2.6 5-FU injections (*p* = 0.440). More suture lyses to titrate IOP (1.1 ± 1.1 versus 0.3 ± 0.5, *p* = 0.023) and less needlings (0.1 ± 0.4 versus 0.4 ± 0.9, *p* = 0.203) were required in the POAG group than in the PEXG group.

### 3.4. Complications

The number and type of complications during and after surgery are displayed in Table 3. Most of the early complications resolved spontaneously; the one case of iris prolapse required revision surgery to reposition the iris. Both cases of late complications (bleb fibrosis and scarring of the sclera flap) required surgical revision and were considered surgical “failure” based on the definitions given above. There was no relevant difference in complication rate or severity between the high and low ET-1 groups.

### 3.5. Surgical Success Rates

**Figure 4:**
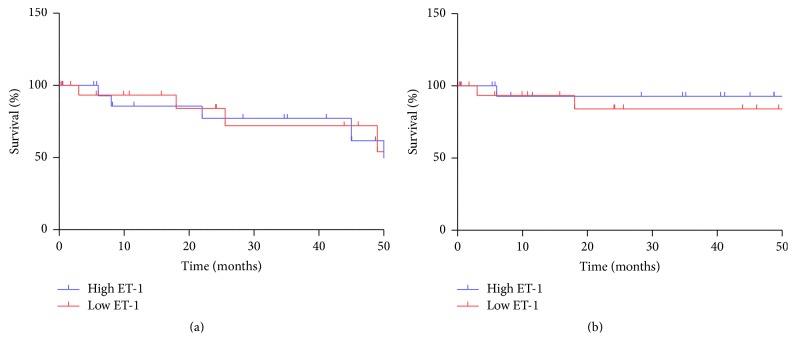
Absolute success rate (a) and survival including relative success (b) over time. Ticks indicate censored data (final follow-up visits to our clinic). There is no difference in overall success of trabeculectomy between patients with high (blue) and low (red) ET-1 concentration in aqueous humor. Both groups have comparable, very good long-term outcomes of surgery.

Absolute success, defined as IOP < 18 mmHg without additional medication or further surgical intervention, was achieved for 90%, 81%, 76%, and 68% at 12, 24, 36, and 48 months after surgery. Including relative success, with additional medication to maintain IOP below 18 mmHg or revision surgery due to complications, but not additional pressure lowering surgery, success rates were 93% at 12 months and remained at 89% for the entire follow-up time thereafter. There was no difference in surgical success between patients with high and those with low ET-1 concentrations in aqueous humor (*p* = 0.715 for absolute success and *p* = 0.953 for relative success, log-rank (Mantel-Cox) test; Figure 4) nor between POAG and PEX glaucoma (8% versus 9.1% failure rate, data not shown).

## 4. Discussion

Endothelin-1 has repeatedly been shown to be increased in different forms of open angle glaucoma [22–24]. While still controversially discussed, there is accumulating evidence for a physiological and/or pathophysiological role of the peptide in the regulation of IOP via effects on the contractility of ciliary muscle and trabecular meshwork. Evidence from animal models suggests that increases in ET-1 concentration may contribute to increases in IOP by eliciting a smooth muscle like contraction of the trabecular meshwork and thereby raising outflow resistance through the tissue [8, 9]. For human tissue, no direct observation of native trabecular meshwork contractility has been published yet, but experimental evidence with cultured human TM cells suggests that the proposed mechanism may be relevant to IOP regulation in glaucoma patients [19, 20]. Interfering with ET-1 signaling has therefore been proposed as a potential means of lowering IOP pharmacologically [7, 17].

In a recent study by our group, a statistically highly significant, albeit numerically small, correlation was shown between IOP and ET-1 in aqueous humor of glaucoma and cataract patients [16]. While this study did not allow establishing a causative relationship between the two parameters, an influence of ET-1 on IOP regulation via effects on the contractile state of the TM appears plausible. Assuming this mechanism is correct, bypassing the TM surgically, that is, by trabeculectomy, should in theory abolish, or at least diminish, the association between ET-1 in aqueous humor and IOP.

Our analysis of the postoperative IOP development of patients with known ET-1 concentrations shows this expected loss of association very well: while before surgery there was a significant difference in IOP between a patient group with high and a group with low ET-1 concentrations, no difference in IOP was observed at any point after surgery (Figures 1 and 2). Similarly, when comparing POAG patients with PEXG patients, who had higher ET-1 concentrations and higher IOP before surgery, there was no difference in IOP after surgery. These observations might therefore be indirect evidence for the proposed effect of ET-1 on human TM contractility and IOP regulation.

Since the major site of outflow resistance, the TM, is bypassed by trabeculectomy, the resulting long-term IOP is mainly determined by the amount of aqueous being resorbed by the conjunctival bleb that forms after surgery. Careful maintenance of a wide, thin-walled, hypovascular filtering bleb is therefore one of the major goals of postoperative care [25].

Failure to control IOP after trabeculectomy is most often the result of excessive scarring of the conjunctival wound. The drained aqueous fluid has been shown to contain various profibrotic growth factors, exposure to which leads to thick, fibrotic tissue with limited capacity for aqueous resorption [26, 27]. Bleb fibrosis was a common occurrence in the early days of trabeculectomy; this limitation has in recent years been partially overcome by the use of antifibrotics like Mitomycin C (MMC) during surgery and 5-fluorouracil (5-FU) during the early postsurgical phase. Both substances have greatly improved the surgical outcome of trabeculectomy [28, 29]. However, long-term results still show a cumulative failure rate of TE of up to 10% per year [30–32]. Moreover, MMC and 5-FU are associated with a number of unwanted side effects due to the fact that their mechanisms of action are not limited to the scarring tissue but affect the corneal epithelium, limbal, and conjunctival stem cells as well [33, 34]. There is a great need to better understand the scarring process in order to target it more specifically and with fewer side effects.

Likely the most important growth factor involved in bleb fibrosis is transforming growth factor beta 2 (TGF-*β*_2_), which has been shown to be the major isoform in aqueous humor, and which is found in increased concentrations in the aqueous humor of patients with bleb fibrosis. However, not all patients with fibrotic blebs also have high concentrations of TGF-*β*_2_ [26, 35]. Other growth factors must also contribute to the scarring process.

ET-1 has been shown to be involved in fibrosis in various organ systems. It is a major pharmaceutical target in primary pulmonary hypertension as well as systemic sclerosis [36]. It has also been shown to contribute to fibrosis in the heart, liver, and kidney. In particular, ET-1 can facilitate the induction of a myofibroblast phenotype in various “precursor” cells, like fibroblasts, stellate cells of the liver, pericytes, monocytes, and epithelial/endothelial cells [21]. Moreover, ET-1 can work both in conjunction with and as a downstream effector of TGF-*β* [37, 38].

While the effects of ET-1 on conjunctival or Tenon's fibroblasts have never been investigated* in vitro* or* in vivo*, it appears reasonable to assume that similar mechanisms as described in various other tissues could also be found in the eye. In fibrotic filtering blebs after trabeculectomy as well as in the hypertrophic capsules surrounding failed glaucoma drainage devices myofibroblasts are the major cell type contributing to the deposition of collagen and fibronectin, two of the main constituents of fibrotic tissue [27, 39]. Moreover, it has been shown that contractility facilitated through Rho-kinase (ROCK) dependent mechanisms is a prerequisite for myofibroblast transdifferentiation in Tenon's fibroblasts and that inhibiting ROCK by specific inhibitors as well as statins prevents this change of phenotype [40, 41]. Since ET-1 has also been shown to act through ROCK in other ocular tissues, increased concentrations of the peptide in aqueous may well contribute to postoperative scarring of filtering blebs [18, 42, 43]. Our chart review therefore also aimed to assess bleb fibrosis or precursors thereof in relation to ET-1 concentrations.

Our results show no indication for an increased tendency for fibrosis in the high ET-1 group. There were only two patients with clear signs of fibrosis: one scarred bleb in the low ET-1 group and one scarred scleral flap in the high ET-1 group, which was deemed to be an “unusual finding” according to the surgeon.

In our study, all patients received the same amount of MMC (0.1 mL at 0.2 mg/mL for 5 minutes) during surgery. Therefore scarring was less likely to occur. Differences in fibrotic propensity might have shown indirectly, however, for example, in the number of 5-FU injections needed in the immediate aftermath of trabeculectomy. Application of 5-FU is typically used based on clinical signs of inflammation and impending early bleb fibrosis like hyperemia of the conjunctiva above the bleb and “cork screw vessels.” Our data, however, show no difference in the use of 5-FU between the groups. Similarly, there was no difference in the average number of suture lyses needed to titrate IOP to desirable levels nor in the number of needlings required to detach the bleb walls from the sclera (Figure 3).

Another indirect measure for the onset of fibrosis might be the need for additional glaucoma medication in order to control IOP. As scarring progresses the amount of aqueous resorbed through the bleb walls drops, making the need for other means of controlling IOP more likely. In our study there was no difference in the number and timing of new glaucoma medications.

There are a number of limitations to our study, like its retrospective design, or the rigid selection criteria. The latter were necessary for the initial study in order to avoid any interference from systemic disorders with increased ET-1 concentrations in blood plasma. Such diseases might have masked the hypothesised correlation between ET-1 in aqueous and IOP. We therefore only included patients that were in excellent health. Patients had no potentially confounding systemic disorders (e.g., cardiovascular and vasospastic disorders, diabetes mellitus, pulmonary diseases, or solid tumors). Similarly, because manipulation of the eye might affect intraocular ET-1 concentrations, only patients without any other ophthalmological diseases, prior ocular surgery, or trauma were included (for further information see [16]). Our patients are therefore not a representative sample of glaucoma patients, who are typically afflicted with one or more of the diseases mentioned above. While this selectivity was quite necessary for the previous study, the results of this retrospective investigation may suffer from a selection bias and can therefore not be generalized.

On the other hand, our general results like age of the included patients, overall success rate, long-term IOP control, and use of additional medication are in good agreement with larger, more representative study populations. The Advanced Glaucoma Intervention Study (AGIS) and the Collaborative Initial Glaucoma Treatment Study (CIGTS), for instance, had similar baseline characteristics: 67.0 and 58.1 years of age versus 63.3 in our study; presurgical IOP of 25.5 and 27.4 mmHg versus 27.5 mmHg, respectively; presurgical number of medications of 2.7 in the AGIS versus 2.8 in our study [44, 45].

The sample size may also affect interpretation of the results. With only 18 patients per group, our study is greatly underpowered to detect small differences. Also, due to its retrospective nature not all patients were present for all selected time points of analysis. However, since the outcome in both groups are almost identical with regard to number of adjunct medications, 5-FU injections, suture lyses, and needlings, the required sample size to determine a significant difference would have been excessively large. Moreover, such small differences would most likely not be clinically relevant.

More importantly, nothing is currently known about the dynamics of ET-1 concentrations in aqueous humor. We only determined ET-1 levels once, in samples collected during the initial surgery. One cannot safely assume that ET-1 concentrations remain static or that they are unaffected by ocular or systemic conditions or medication. They might even have been altered by the surgery itself. Since we only included otherwise healthy subjects, systemic effects on ET-1 should have been minimal (as shown by the lack of difference in systemic ET-1 levels between groups). Yet, we cannot rule out that ET-1 levels in aqueous humor are highly dynamic and the single sample is not representative of long-term ET-1 concentrations. Since it is difficult to sample aqueous humor on a regular basis, we have to contend with this limitation.

In conclusion, our results demonstrate that a potential regulatory role of ET-1 on IOP through actions on the trabecular meshwork is effectively abolished by shunting this tissue. In our small sample of otherwise healthy glaucoma patients, increased concentrations of Endothelin-1 in the aqueous humor did not influence the surgical outcome of standard trabeculectomy with MMC during up to four years of follow-up. This does not preclude a long-term effect of very high concentrations in aqueous humor or of systemically increased ET-1 on wound healing and bleb fibrosis. Further laboratory studies on the interactions of ET-1 with conjunctival and Tenon's fibroblasts as well as prospective clinical studies with larger, more representative patient groups may be useful to elucidate potential effects of ET-1 on trabeculectomy outcome.

## Figures and Tables

**Table 1:** Patient characteristics.

	All patients	POAG	PEXG	Uncorrected *p* values
Number included	36	25	11	
Age (years)	63.3 (±11.9)	62.2 (±13.9)	65.9 (±4.8)	*p* = 0.396
Gender (m/f)	17/20	13/12	3/8	*p* = 0.277
Study eye (OD/OS)	13/23	11/14	2/9	*p* = 0.259
Last IOP before surgery (mmHg)	27.5 (±8.5)	25.2 (±5.8)	32.6 (±11.3)	**p** ** = 0.013**
Number of medications	2.8 (±0.9)	2.9 (±0.8)	2.5 (±1.1)	*p* = 0.199
ET-1 in aqueous humor (pg/mL)	6.5 (±2.8)	5.9 (±2.9)	7.8 (±1.9)	*p* = 0.062

**Table 2:** General characteristics of study groups.

	High ET-1	Low ET-1	
ET-1 in aqueous humor (pg/mL)	8.7 (±2.1)	4.4 (±1.5)	
Last IOP before surgery (mmHg)	31.2 (±9.6)	23.8 (±5.2)	**p = 0.007**
Age (years)	63.4 (±12.7)	63.3 (±11.4)	*p* = 0.996
Gender (m/f)	11/7	5/13	*p* = 0.092
Study eye (OD/OS)	3/15	10/8	*p* = 0.035
Number of medications	2.8 (±1.0)	2.7 (±0.8)	*p* = 0.857
Diagnosis (POAG/PEXG)	10/8	15/3	*p* = 0.146
Follow-up (months)	30.8 (±19.9)	27.8 (±21.4)	*p* = 0.661

**Table 3:** Complications.

Complication	High ET-1	Low ET-1
Corneal erosion	2	2
Iris prolapse	1	0
Hypotony	1	0
Choroidal detachment	0	1
Fibrosis of sclera flap	0	1
Bleb fibrosis	1	0
